# Superconductivity emerging from a stripe charge order in IrTe_2_ nanoflakes

**DOI:** 10.1038/s41467-021-23310-w

**Published:** 2021-05-26

**Authors:** Sungyu Park, So Young Kim, Hyoung Kug Kim, Min Jeong Kim, Taeho Kim, Hoon Kim, Gyu Seung Choi, C. J. Won, Sooran Kim, Kyoo Kim, Evgeny F. Talantsev, Kenji Watanabe, Takashi Taniguchi, Sang-Wook Cheong, B. J. Kim, H. W. Yeom, Jonghwan Kim, Tae-Hwan Kim, Jun Sung Kim

**Affiliations:** 1 Center for Artificial Low Dimensional Electronic Systems, Institute for Basic Science, Pohang, Korea; 2 Department of Physics, Pohang University of Science and Technology, Pohang, Korea; 3 Department of Materials Science and Engineering, Pohang University of Science and Technology, Pohang, Korea; 4 Laboratory for Pohang Emergent Materials, Pohang Accelerator Laboratory, Pohang, Korea; 5 Max Planck POSTECH/Korea Research Initiative, Pohang, Korea; 6 Department of Physics Education, Kyungpook National University, Daegu, Korea; 7 Korea Atomic Energy Research Institute (KAERI), Yuseong-gu, Daejeon Korea; 8 M.N. Mikheev Institute of Metal Physics, Ural Branch, Russian Academy of Sciences, Ekaterinburg, Russia; 9 NANOTECH Centre, Ural Federal University, Ekaterinburg, Russia; 10 Research Center for Functional Materials, National Institute for Materials Science, Tsukuba, Japan; 11 International Center for Materials Nanoarchitectonics, National Institute for Materials Science, Tsukuba, Japan; 12 Rutgers Center for Emergent Materials and Department of Physics and Astronomy, Rutgers University, Piscataway, NJ USA; 13 Asia Pacific Center for Theoretical Physics (APCTP), Pohang, Korea

**Keywords:** Superconducting properties and materials, Surfaces, interfaces and thin films, Two-dimensional materials

## Abstract

Superconductivity in the vicinity of a competing electronic order often manifests itself with a superconducting dome, centered at a presumed quantum critical point in the phase diagram. This common feature, found in many unconventional superconductors, has supported a prevalent scenario in which fluctuations or partial melting of a parent order are essential for inducing or enhancing superconductivity. Here we present a contrary example, found in IrTe_2_ nanoflakes of which the superconducting dome is identified well inside the parent stripe charge ordering phase in the thickness-dependent phase diagram. The coexisting stripe charge order in IrTe_2_ nanoflakes significantly increases the out-of-plane coherence length and the coupling strength of superconductivity, in contrast to the doped bulk IrTe_2_. These findings clarify that the inherent instabilities of the parent stripe phase are sufficient to induce superconductivity in IrTe_2_ without its complete or partial melting. Our study highlights the thickness control as an effective means to unveil intrinsic phase diagrams of correlated van der Waals materials.

## Abstract (web summary)

Superconductivity often appears due to suppression of competing electronic orders. Here, the authors present a contrary example showing a superconducting dome inside the parent phase with a stripe charge order in IrTe_2_ nanoflakes and identify their unusual superconducting properties.

## Introduction

**Fig. 1:**
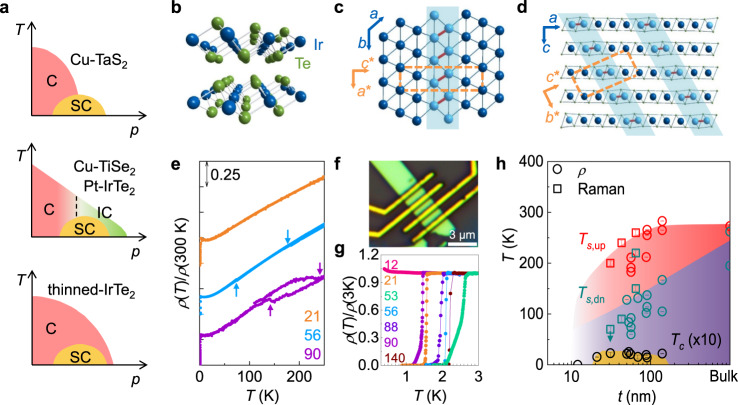
Structure and phase diagram of IrTe_2_ nanoflakes. **a** Schematic phase diagrams of TMDCs as a function of control parameter *p*, showing commensurate (C), incommensurate charge order (IC), and superconductivity (SC). Three different types of dome-shaped superconducting phase diagram, where the dome lies at the centre of a presumed quantum critical point (top), near the C-IC transition line (middle), or well inside the parent order (bottom). **b** Crystal structure of IrTe_2_. **c**, **d** Schematic illustrations of the stripe order in IrTe_2_ below *T*_*s*_. The Ir-Ir dimerization (red) with a modulation vector **q** = ($\frac{1}{5}$,0,$\frac{1}{5}$) is depicted (blue shade) on the triangular Ir layer (**c**) and across the stacked layers (**d**). The crystallographic axes for the high-*T* (*a*, *b*, and *c*) and the low-*T* (a^*^, b^*^, and c^*^) structures are shown, together with the unit cell of the stripe phase (orange box). **e** Temperature dependence of the normalised resistivity *ρ*(*T*)/*ρ*(300 K) for IrTe_2_ crystals with thickness (*d*) of 21, 56, and 90 nm. For clarity, *ρ*(*T*)/*ρ*(300 K) curves are offset vertically. Transition temperatures *T*_*s*,up_ and *T*_*s*,dn_ are determined (arrows) in opposite temperature sweeps. **f** Optical microscope image of a 56-nm-thick IrTe_2_ crystal. **g** *ρ*(*T*)/*ρ*(3 K) curves for IrTe_2_ crystals with *d* = 12–140 nm. **h** Phase diagram of IrTe_2_ nanoflakes as a function of thickness *d*, obtained by transport (circle) and Raman spectroscopy (square) measurements. The transition temperatures *T*_*s*,up_ (red) and *T*_*s*,dn_ (blue) during warming and cooling are plotted with the superconducting transition temperature *T*_*c*_ (black), scaled by a factor of 10 for clarity. The error bars from the resistivity and Raman spectroscopy are defined by the width of the corresponding resistive transitions and the temperature step of 5 K between the measurements, respectively.

Transition metal dichalcogenides (TMDCs) provide a prototypical quasi-two-dimensional system, possessing various electronic instabilities to periodic charge modulations^1^. These instabilities often induce complex charge-density-wave (CDW) phases with different commensurability conditions^2–5^, sometimes associated with Mott-^2^ or excitonic insulating^6^ phases. Upon chemical doping^4,5,7,8^, these phases are commonly driven into a superconducting phase, resulting in a characteristic dome-shaped phase diagram, reminiscent of those found in other unconventional superconductors^9–11^ (Fig. 1a). Understanding the complex interplay between charge ordering and superconducting instabilities in TMDCs is a long standing issue, which has often been hampered by presence of quenched disorders, introduced in chemical doping. Recently, utilising the weak van der Waals (vdW) coupling between layers, TMDCs were found to be thinned down to atomic length scale^12^, comparable with the coherence lengths of their various electronic orders. This offers another effective way to tune stability or properties of the competing phases, as demonstrated for 1*T*-TaS_2_^13–15^ and NbSe_2_^16,17^, in which distinct thickness dependence of the transition temperatures is observed for superconducting and CDW phases.

IrTe_2_ is one of the TMDCs in vdW structure (Fig. 1b), showing a similar dome-shaped superconducting phase diagram^18–23^. IrTe_2_ undergoes a stripe charge ordering transition at *T*_*s*_ ~ 260 K, and by suppressing it with *e*.*g*. chemical doping^18–23^ the superconducting phase eventually appears, similar to TMDCs hosting the parent CDW orders^4,5,7,8^. The stripe order in IrTe_2_, however, is accompanied by the first-order structural transition involving in-plane Ir–Ir dimerization and interlayer Te–Te depolymerisation^24,25^, which forms stripe patterns with a predominant period of 5*a*_0_ (*a*_0_, the *a* axis lattice constant) as depicted in Fig. 1c and d^19,26^. No clear evidence of gap opening in the Fermi surface (FS) is observed^27,28^, unlike the typical CDW gap formation in TMDCs^29^. Instead, FS reconstruction to the so-called cross-layer two-dimensional (2D) state^30,31^ occurs due to suppression of the density of states (DOS) in the planes of Ir–Ir dimers running across the vdW gaps. These aspects suggest that the relationship between the stripe and superconducting orders in IrTe_2_ may differ significantly from those of other TMDCs, as indicated by recent discoveries on the superconductivity in quenched or thinned IrTe_2_ crystals^32–34^. Here using Raman spectroscopy, scanning tunnelling microscopy, and transport property measurements, we found that the parent stripe phase encompasses the whole superconducting dome in the thickness-dependent phase diagram (Fig. 1a). This unusual coexistence of the stripe and superconducting orders significantly increases the interlayer coherence length and the coupling strength of superconductivity in IrTe_2_ nanoflakes, revealing the collaborative role of the stripe order to the superconductivity in IrTe_2_.

## Results

### Thickness dependent phase transitions

**Fig. 2:**
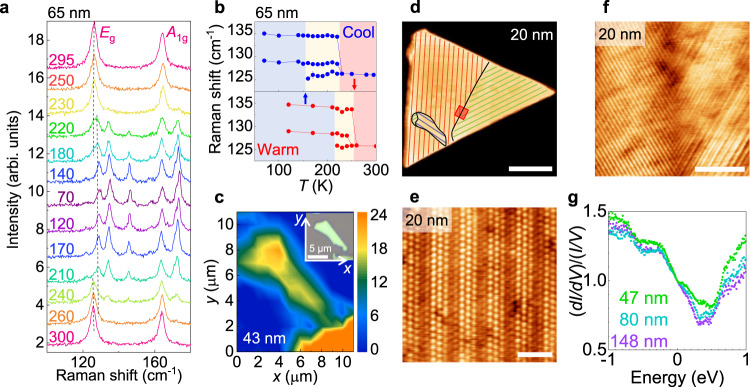
Stripe charge ordering formation in IrTe_2_ nanoflakes. **a**, **b** Raman spectra (**a**) and corresponding temperature dependent Raman frequency (**b**) of a 65-nm-thick IrTe_2_ nanoflake at various temperatures during cool-down and warm-up procedures. At *T* < *T*_*s*_, the Raman modes, *E*_g_ at 126 cm^−1^ and *A*_1g_ at 165 cm^−1^, split into multiple peaks as the flake forms the stripe charge order in **a**. The temperature ranges for the normal (red), the stripe (blue), and intermediate coexistence (yellow) phases, are identified in **b**, during cooling (upper panel) and warming (lower panel). Transition temperatures *T*_*s*,dn_ and *T*_*s*,up_ are indicated by the arrows, respectively. **c** Spatial profile of Raman intensity map for 129 cm^−1^ for a 43-nm-thick IrTe_2_ nanoflake at *T* ~ 70 K. Inset: optical microscope image of the flake. **d** Large-scale scanning tunnelling microscopy (STM) image of a 20-nm-thick IrTe_2_ flake at *T* = 85 K (scale bar, 300 nm). The flake has only stripe-phase charge-ordered domains, illustrated by red, green, and blue lines. Black lines indicate domain boundaries between three equivalent stripe-phase charge-ordered domains. **e** Atomically resolved STM image of **d **at *T* = 85 K representing a uniform striped area with 5 × 1 surface reconstruction (scale bar, 2 nm). **f** Zoomed-in STM image of two charge-ordered phases indicated by red square in **d** showing that the two phases coexist at the boundary (scale bar, 20 nm). **g** Scanning tunnelling spectroscopy (STS) spectra at *T* = 85 K taken on IrTe_2_ nanoflakes with *d* = 47, 80, and 148 nm, as indicated in the plot.

**Fig. 3:**
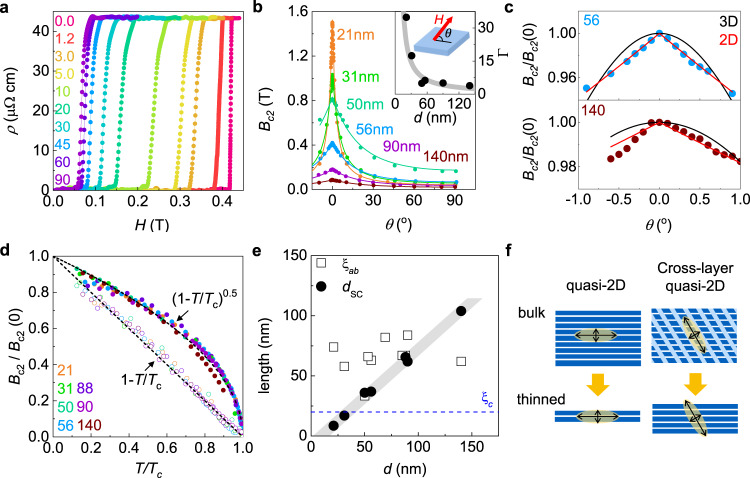
Two dimensional superconductivity of IrTe_2_ nanoflakes. **a**, Magnetic field dependence of *ρ*(*H*) of a 56-nm-thick IrTe_2_ nanoflake, measured with different field orientations *θ* at *T* = 0.35 K. **b**, Upper critical field *B*_*c*2_ as a function of field angle *θ* for IrTe_2_ nanoflakes with different thickness (*d*) at *T* = 0.35 K, together with the fit (solid line) to the 2D Tinkham model. Inset: the anisotropy factor ${{\Gamma }}={B}_{c2}^{ab}$/${B}_{c2}^{c}$ as a function of *d*, following 1/*d* dependence (grey line). Schematic illustration shows the field orientation *θ*. **c**, Angle dependence of *B*_*c*2_(*θ*) of IrTe_2_ nanoflakes with *d* = 56 and 140 nm at *T* = 0.35 K. Good agreement with the 2D Tinkham model (red), rather than the 3D Ginzburg-Landau model (black), confirms the 2D superconductivity. **d**, Normalised *B*_*c*2_/*B*_*c*2_(0) as a function of *T*/*T*_*c*_ for IrTe_2_ nanoflakes with different *d*. All data collapse into dashed lines described by 1 − *T*/*T*_*c*_ and ${(1-T/{T}_{c})}^{1/2}$ for *B*∥*c* (open circles) and *B*∥*ab* (solid circles), respectively. **e**, Ginzburg-Landau coherence length *ξ*_*ab*_ (square) and the effective superconducting thickness *d*_SC_ (circle) as a function of *d*. *ξ*_*ab*_ is nearly independent of *d*, whereas *d*_SC_ grows linearly with *d* (*d*_SC_ ~ 0.8*d*) and exceeds *ξ*_*c*_ of doped bulk IrTe_2_. **f**, Schematic illustration of the size effect of vdW superconductors. In normal vdW superconductors with a large anisotropy *ξ*_*c*_ ≪ *ξ*_*ab*_, 2D superconductivity appears only for a-few-layer-thick crystals. In IrTe_2_ with a stripe order and the resulting cross-layer quasi-2D state, the increased *ξ*_*c*_ ~ *ξ*_*ab*_ induces 2D superconductivity in relatively thick nanoflakes.

In order to vary the thickness of IrTe_2_, we employed the mechanical exfoliation method of single crystals and obtained thin flakes with thickness (*d*) down to ~10 nm, which show a systematic thickness dependence of the in-plane resistivity *ρ* (Fig. 1e). For the temperature sweeps in both directions, we took a slow cooling rate of ~0.5 K/min, in order to minimise inhomogeneous domain formation of the stripe-charge-ordered and charge-disordered phases, found in rapid-cooled IrTe_2_ crystals^32,33,35^. The temperature dependent *ρ*(*T*) follows a metallic temperature dependence with abrupt changes at *T*_*s*,dn_ and *T*_*s*,up_, due to the first-order stripe ordering transition as found in bulk crystals^24,25^. These resistive anomalies across the transitions, however, become much smaller in size with reducing *d*, and eventually disappears for *d* < 50 nm. This does not mean full suppression of the stripe order in thin nanoflakes, since the size of the resistive anomaly is known to be strongly suppressed by introducing strain, reducing the sample volume, and increasing the cooling rate even in bulk samples^34^. Rather, the stripe order is found to be stable in all the nanoflakes we studied, as discussed below (Fig. 2). At low temperatures, all the nanoflakes, except the thinnest one with *d* = 12 nm, exhibit a superconducting transition as found in the temperature-dependent normalised resistance *ρ*(*T*)/*ρ*(3 K) (Fig. 1g). The superconducting transition temperature, defined as a 50% resistive transition, is *T*_*c*_ = 1.43–2.64 K, somewhat lower than *T*_*c*_ ~ 3 K for the optimally doped bulk IrTe_2_^18,19^, mostly due to the 2D nature as discussed below (Fig. 3). Unlike the stripe charge ordering transition, the superconductivity is found to be quite stable in nanoflakes. The superconducting transition temperatures and widths remain almost the same in different thermal cycling (Supplementary Fig. 7).

The stripe ordering transition of IrTe_2_ nanoflakes is characterised by Raman spectroscopy. For bulk IrTe_2_, two Raman active modes, *E*_g_ at 126 cm^−1^ and *A*_1g_ at 165 cm^−1^, in a trigonal structure (space group $P\bar{3}m1$) split into multiple peaks due to the lowered symmetry and the emergence of a super unit cell for the stripe order below *T*_*s*_^30,36^. Raman spectra taken from the 65-nm-thick nanoflake with sequential decrease and increase of temperature (Fig. 2a) reveal that both *E*_g_ and *A*_1g_ Raman modes at room temperature split into multiple Raman modes at *T* = 70 K, well below *T*_*s*_, consistent with previous studies on the bulk^36^. This behaviour is also shown for the flakes with different thicknesses (Supplementary Fig. 2a and Fig. 3a) and confirms formation of the stripe charge order in our IrTe_2_ nanoflakes.

In IrTe_2_ nanoflakes, however, the temperature dependence of the stripe phase evolution is distinct from the bulk case. We found that below *T*_*s*_ there is an intermediate temperature range (yellow range in Fig. 2b) where the high temperature Raman modes coexist with the low temperature modes, for both temperature sweeps. This contrasts to the abrupt change found in bulk IrTe_2_ with a negligible coexistence range^36^ and indicates macroscopic phase separation of the normal and stripe phases in IrTe_2_ nanoflakes at the intermediate temperature. We defined *T*_*s*,dn_ and *T*_*s*,up_ as the temperatures where the contribution from the normal and stripe phase disappears during the cool-down and warm-up procedures, respectively (Fig. 2b). As the flake thickness decreases, the transition temperatures monotonically decrease, which are in good agreement with those from *ρ*(*T*) (Fig. 1h). In addition, we measured the Raman spectroscopy on IrTe_2_ flakes, cooled down to *T* = 4 K, just above *T*_*c*_ ~ 2 K. For IrTe_2_ flakes with various thicknesses from 10 nm to 174 nm, which cover the whole superconducting dome in the thickness-dependent phase diagram, we observed clear splitting of Raman modes, consistent with the stripe-charge-order formation (Supplementary Fig. 3). These results clearly show that the region of the stripe phase overlaps with the entire superconducting dome in a wide range of thickness.

### Coexistence of stripe order and superconductivity

This phase diagram significantly differs from the doping-dependent phase diagram of bulk IrTe_2_^18–23^. In the bulk case, the stripe and superconducting phases are mutually exclusive, and the coexisting region appears in the very narrow doping range^18–23^. Even in this coexisting phase, two ordered phases are macroscopically separated^22,23^. Such a macroscopic phase separation is also suggested in the super-cooled case^32,33,35^. However, in IrTe_2_ nanoflakes, the stripe phase completely covers the whole regions of the sample, as confirmed by the spatial mapping of Raman signal, taken at ~ 70 K well below *T*_*s*_ (Fig. 2c). The intensity map of the 129 cm^−1^ Raman mode, which is the hallmark of the stripe phase, reveals a strong Raman intensity profile over the entire nanoflake as in the optical microscope image (Fig. 2c inset), while the signal from the normal phase is absent (Supplementary Fig. 2c). This indicates that the stripe phase dominantly prevails in the macroscopic length scale and serves as a normal state for the superconductivity, in contrast to the doped bulk IrTe_2_ case.

The dominant stripe phase formation is further confirmed by scanning tunnelling microscopy (STM) for a representative nanoflake with *d* = 20 nm (Fig. 2d). At room temperature, the hexagonal lattice of top-most Te atoms is clearly resolved by STM in all IrTe_2_ nanoflakes (Supplementary Fig. 4b). When cooled down below the stripe ordering temperature (*T*_STM_ = 85 K < *T*_*s*_), the ultrathin nanoflake develops clear stripe patterns with a period of 5*a*_0_ due to the charge ordering and dimerization of Ir atoms (Fig. 2e), as observed in bulk crystals^25,26^. By scanning over the nanoflake with sufficient spatial resolution (Fig. 2f), we confirm that the whole surface of the flake hosts one or two predominant stripe phases among three energetically equivalent phases (Fig. 2d), and the stripe patterns are often oriented nearly parallel to the long edges of nanoflakes. In thicker nanoflakes (Supplementary Fig. 5a), stripe domain patterns became more complex, similar to bulk IrTe_2_^35^. We note that even with fast cooling at ~ 1 K/sec, neither thin nor thick nanoflakes show the hexagonal phase that is often observed in either super-cooled or doped bulk IrTe_2_^22,35^ and considered to be responsible for the superconductivity. Consistently, in the scanning tunnelling spectroscopy (STS) measurements on IrTe_2_ nanoflakes, we observe similar local DOS over the wide range of thicknesses. In STS spectra (Fig. 2g), obtained on different IrTe_2_ nanoflakes with 47 ≤ *d* ≤ 148 nm, the local spectral features are qualitatively consistent with the total DOS for the stripe phase of bulk IrTe_2_, as estimated using first principle calculations^30^. Our results from STM and Raman spectroscopy provide strong evidence that the superconductivity emerges from the preexisting stripe phase in IrTe_2_ nanoflakes.

### 2D superconductivity

Now we focus on the effect of the underlying stripe order on the superconducting properties. To address this issue, we investigated the upper critical field *B*_*c*2_ of each nanoflake as a function of field orientation below *T*_*c*_. Figure 3a shows the resistivity *ρ*(*H*) curves of a representative nanoflake with *d* = 56 nm, collected at *T* = 0.35 K under a magnetic field, for which the angle *θ* is defined with respect to the *ab* plane. The anisotropy of *B*_*c*2_, ${{\Gamma }}={B}_{c2}^{ab}$/${B}_{c2}^{c} \sim 5.3$ for *d* = 56 nm, becomes stronger with lowering *d* and reaches up to Γ ~ 38 for *d* = 21 nm (Fig. 3b), an order of magnitude larger than Γ ~ 2 of doped bulk IrTe_2_^37^. This large increase in Γ with moderate changes in *T*_*c*_ (Fig. 1g) and the in-plane coherence length *ξ*_*ab*_(0) (Fig. 3e) can only be explained by 2D superconductivity. In the Tinkham model of 2D superconductivity^38^, the angle dependent *B*_*c*2_(*θ*) at the zero-temperature limit is described by $| {B}_{c2}(\theta )\sin \theta /{B}_{c2}^{c}| \,+ {({B}_{c2}(\theta )\cos \theta /{B}_{c2}^{ab})}^{2}=1$, where ${B}_{c2}^{ab}= (\sqrt{12}{{{\Phi }}}_{0})$/(2*πξ*_*ab*_(0)*d*_SC_) and ${B}_{c2}^{c}={{{\Phi }}}_{0}$/(2*πξ*_*ab*_(0)^2^) (Φ_0_, a flux quantum). Thus by reducing the effective thickness of the superconducting layer *d*_SC_, Γ becomes large with a constant *ξ*_*ab*_(0), and a discontinuous cusp in the *B*_*c*2_(*θ*) curve near *θ* = 0^∘^ is expected. These predictions are distinct from those of the Ginzburg-Landau model for anisotropic three-dimensional (3D) superconductors^38^, as described by *B*_*c*2_(*θ*) = *B*_*c*2_(0^∘^)/$\sqrt{{{{\Gamma }}}^{2}{\sin }^{2}\theta +{\cos }^{2}\theta }$. All *B*_*c*2_(*θ*) curves exhibit a clear cusp near *θ* = 0^∘^ and are successfully fitted by the 2D Tinkham model rather than the anisotropic 3D model (Fig. 3b and Supplementary Fig. 9). For *d* = 140 nm, the thickest sample, *B*_*c*2_(*θ*) slightly deviates from the 2D model, but still far from 3D model (Fig. 3c). These results demonstrate that IrTe_2_ nanoflakes with *d* ≤ 140 nm clearly show the 2D superconductivity.

The temperature dependence of *B*_*c*2_(*T*) under in-plane (*B*∥*ab*) and out-of-plane (*B*∥*c*) magnetic fields further confirms the 2D superconductivity of IrTe_2_ nanoflakes. For seven samples with different *d*’s, we determined *B*_*c*2_(*T*) by taking 50% of the resistive transition as a function of the normalised temperature *t* = *T*/*T*_*c*_ (Fig. 3d and Supplementary Fig. 8). The out-of-plane ${B}_{c2}^{c}(t)$ is almost the same, following the linear dependence (Fig. 3d), as observed in the doped bulk sample^37^. The in-plane ${B}_{c2}^{ab}(t)$ increases strongly with lowering *d*, but the normalised *B*_*c*2_(*t*)/*B*_*c*2_(0) curves for all samples collapse into a single curve following the 2D Ginzburg-Landau model^38^, ${B}_{c2}^{ab}(t)=\frac{{{{\Phi }}}_{0}}{2\pi }\frac{\sqrt{12}}{{\xi }_{ab}{d}_{{\rm{SC}}}}{\left(1-t\right)}^{1/2}$. Using *ξ*_*ab*_(0), estimated from the observed ${B}_{c2}^{c}(0)$, we obtained *d*_SC_ is ~ 80% of the measured thickness *d* (Fig. 3e). Considering that 2D superconductivity is induced at *d* ( ~ *d*_SC_) smaller than the out-of-plane coherence length *ξ*_*c*_, *i.e*. *d* < *ξ*_*c*_, we conclude that *ξ*_*c*_(0) of IrTe_2_ nanoflakes should be larger than the maximum value of *d*_SC_ ~ 100 nm, obtained in experiments (Fig. 3e), This value is significantly larger than the typical *ξ*_*c*_(0) ~ 25 nm of doped IrTe_2_ bulk samples^37^, and comparable to the in-plane coherence length *ξ*_*ab*_(0) ~ 70 nm^39^ (Fig. 3e). These findings clearly indicate that the superconducting characteristics of IrTe_2_ nanoflakes are distinct from those of the doped IrTe_2_.

### Characteristics of superconductivity coexisting stripe order

The drastically increased *ξ*_*c*_(0) in IrTe_2_ nanoflakes is a consequence of the coexisting stripe order. In anisotropic superconductors, the interlayer coherence length *ξ*_*c*_(0) is determined by the superconducting gap (Δ_SC_) and the Fermi velocity (${v}_{{\rm{F}}}^{c}$), *i.e*. ${\xi }_{c}(0)\propto {v}_{{\rm{F}}}^{c}$/Δ_SC_. Assuming a similar Δ_SC_, *ξ*_*c*_(0)/${\xi }_{ab}(0)\approx {v}_{{\rm{F}}}^{c}$/${v}_{{\rm{F}}}^{ab}\gtrsim 1$ seems incompatible with the vdW structure of IrTe_2_. This however can be explained by considering the coexisting stripe order. In the stripe phase of IrTe_2_, Ir-Ir dimerization and Te-Te depolymerisation produce conducting planes between the dimer planes, running across the vdW gaps (Fig. 1d). This cross-layer 2D conducting state^30^ affects the electronic structure such that the interlayer ${v}_{{\rm{F}}}^{c}$ is even larger than the in-plane ${v}_{{\rm{F}}}^{ab}$, which greatly increases *ξ*_*c*_(0) (Fig. 3f). Unlike the conventional vdW superconductors in which 2D superconductivity can only be induced in a-few-layer-thick crystals, IrTe_2_ hosts 2D superconductivity in the relatively thick crystals due to the microscopically coexisting stripe order.

**Fig. 4:**
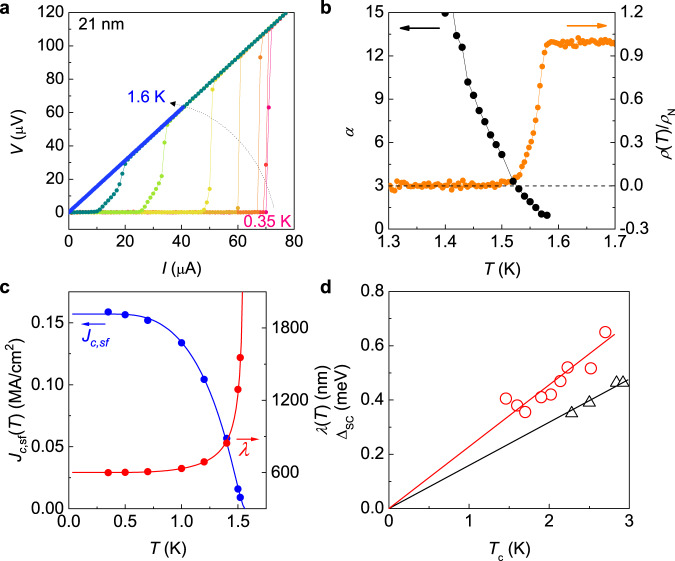
Strong superconducting coupling in IrTe_2_ nanoflakes. **a** Current-voltage (*IV*) characteristics at various temperatures for a representative IrTe_2_ nanoflake with *d* = 21 nm. **b** Temperature dependence of the normalised resistivity and the exponent *α* for a 21-nm-thick IrTe_2_ nanoflake. Exponent *α* is determined from the power-law behaviour *V* ∝ *I*^*α*^ in the *IV* curves, as expected by BKT transition. **c** Critical current density (blue) and the penetration depth (red) as a function of temperature for a 21-nm-thick IrTe_2_ nanoflake. Solid lines are the fits to the self-critical-current model described in the text. **d** Superconducting gap Δ_SC_ as a function of *T*_*c*_, extracted from the critical current density for IrTe_2_ nanoflakes with different *d* (red). The slope of their linear dependence, corresponding to the superconducting gap ratio, 2Δ_SC_/*k*_B_*T*_*c*_ = 5.3, is much larger than the case of doped bulk IrTe_2_ (black) from refs. ^37,42^. This difference confirms the strong coupling nature of the superconductivity in IrTe_2_ nanoflakes.

The distinct superconducting nature in IrTe_2_ nanoflakes is also found in their superconducting gap as compared to the doped bulk. Figure 4a presents the current-voltage (IV) characteristics at different temperatures for a representative nanoflake with *d* = 21 nm. Near *T*_*c*_ ≈ *T*_BKT_, they follow Berezinskii-Kosterlitz-Thouless transition for 2D superconductivity (Fig. 4b), in which the exponent *α* extracted from *V* ∝ *I*^*α*^ crosses *α* = 3 at *T*_BKT_. Well below *T*_*c*_, the self-field critical current density *J*_*c*,s*f*_(*T*) can be obtained from the IV characteristics with variation of temperature, which is proportional to temperature dependent London penetration depth *λ*(*T*) for *d* ≪ *λ*^40,41^. From the critical current *I*_*c*_(*T*), at which the measured voltage jumps due to the superconducting-to-normal transition, we obtained the corresponding *J*_*c*,s*f*_(*T*) and *λ*(*T*) curves (Fig. 4c and Supplementary Fig. 10), which can be nicely reproduced by the fit based on BCS theory using Δ_SC_ ≈ 0.38 meV. The superconducting gap (Δ_SC_), taken from IrTe_2_ nanoflakes with different thicknesses, varies linearly with their *T*_*c*_ with a superconducting gap ratio of 2Δ_SC_/*k*_B_*T*_*c*_ ~ 5.3, which is much larger than the BCS value of 3.53 and 2Δ_SC_/*k*_B_*T*_*c*_ ~ 3.7 of doped bulk IrTe_2_^37,42^ (Fig. 4d). Thus, superconductivity in IrTe_2_ nanoflakes is in the strong coupling regime, whereas that of doped bulk IrTe_2_ is in the weak coupling regime.

## Discussion

Our findings unequivocally emphasise that the superconductivity in IrTe_2_ nanoflakes, which emerges from the preexisting stripe order, is highly distinct from that in doped bulk IrTe_2_. On pristine IrTe_2_ bulk or surface, the 5*a*_0_ stripe phase undergoes multiple transitions to other nearly-degenerate stripe phases that have different periods such as 8*a*_0_ and 6*a*_0_, and also a honeycomb phase^35,43–45^. The complex stripe ordering formation is a result of subtle balance between local interactions of Ir-Ir dimerization and Te-Te depolymerisation. These incipient instabilities and the resulting strong electron-lattice coupling of the parent 5*a*_0_ stripe phase can facilitate pairing interaction for superconductivity in a proper condition and thereby enhancing the superconducting coupling strength as observed in IrTe_2_ nanoflakes. This coexisting phase of stripe and superconducting orders, however, cannot be accessed by chemical doping, *e.g*. Pt doping at the Ir sites. A few % of doping directly perturbs Ir dimerization and melts the stripe order to a quasi-periodic hexagonal order^18,22^. In this case, the onset of superconductivity coincides with disorder-induced melting of the parent order^23^, reminiscent of other TMDCs such as Cu-doped TiSe_2_^3–5^.

In contrast, the thickness control of IrTe_2_ tunes the stripe order without introducing quenched disorders. The stripe order in IrTe_2_ may be mildly suppressed by the thinning-induced out-of-plane elongation^13,46^ or the substrate-induced in-plane tensile strain as opposite to the pressure effect enhancing *T*_*s*_^24^. Typically, the thinning-induced out-of-plane elongation of Δ*c*/*c* ~ 0.1%, as found in TaS_2_^13,46^ and the substrate-induced in-plane strain of Δ*a*/*a* ~ 0.1–0.3% (Supplementary Note 6) are expected in the thinned IrTe_2_, where *a* and *c* are the in-plane and out-of-plane lattice constants, respectively. A recent study on a strained IrTe_2_ single crystals^47^ revealed that only ~ 0.1% of tensile strain induces the transition between the stripe-charge-ordered phases with different periods of 5*a*_0_ and 6*a*_0_, which significantly modifies the electronic structures. Our first principle calculations for electron-phonon coupling constant *λ*_ep_ of the 5*a*_0_ stripe phase show that the in-plane tensile strain is more effective to drive the system to the structural instability and to enhance *λ*_ep_ than the out-of-plane elongation, which increases *T*_*c*_ by a factor of ~ 3 (Supplementary Table 1). While the corresponding critical strain is much larger in calculations and also the calculated *T*_*c*_ remains lower than the measured *T*_*c*_ ~ 2 K, these observations imply that the stripe phase of IrTe_2_ is intrinsically in close proximity to the superconducting phase, which can be accessed by reducing thickness or by thermal quenching^32,33^. It has been known that, in the vicinity of full charge order melting, strong electron-phonon coupling^48,49^ can play an important role to promote the superconductivity, as found in the charge-ordered organic metals^50,51^. This appears to be consistent with the enhanced superconducting gap ratio, found in IrTe_2_ flakes (Fig. 4). Our results unveil the collaborating relationship, rather than the competing one, between the parent stripe and the superconducting orders in IrTe_2_, highlighting IrTe_2_ as a unique example among superconducting TMDCs. Further investigations, for examples, scanning tunnelling microscopy and spectroscopy as well as Raman spectroscopy below the *T*_*c*_ are highly desirable to identify the local superconducting gap structure along the charge modulation patterns and the electron-phonon coupling, which would elucidate the interplay of the stripe-charge-order and superconductivity in IrTe_2_.

## Methods

### Single crystal growth and bulk properties

IrTe_2_ single crystals were synthesised using the Te-flux method^31^. Ir and Te powders were mixed in a stoichiometric ratio Ir:Te = 1:4, heated to 1160 ^∘^C for 1 day as sealed in a quartz ampoule, and then cooled. The crystallinity and stoichiometry were confirmed by X-ray diffraction and energy-dispersive X-ray spectroscopy.

### Exfoliation and fabrication of nanoflakes

We used mechanical exfoliation of bulk single crystals to obtain thin nanoflakes of IrTe_2_ on top of a Si/SiO_2_ substrate that had been pre-cleaned in acetone, 2-propanol, and deionised water, then treated by oxygen plasma (O_2_ = 10 sccm, *P* ~ 100 mTorr) for 5 min. All cleaving and handling were done in inert atmosphere (H_2_O < 0.1 ppm, O_2_ < 0.1 ppm) of pure Ar gas except the atomic force microscopy (AFM) measurements. In some cases, a thin h-BN crystal was subsequently transferred onto the IrTe_2_ nanoflake in Ar atmosphere. We found that the optical contrast, the AFM thickness, and Raman spectra were unchanged even after 1 week in ambient conditions (Supplementary Fig. 1), indicating that the nanoflakes are stable in ambient conditions. To fabricate devices for electrical measurements, we used conventional e-beam lithography to pattern electrodes on top of IrTe_2_ nanoflakes with metal deposition of Cr(10 nm)/Au(50 nm). The optical microscope image for the typical device is shown in Fig. 1f.

### Transport property measurements

Transport measurements were performed in a cryogenic ^3^He refrigerator equipped with a superconducting vector magnet (9/2/2 T). Each measurement wire was filtered by a room-temperature *π* filter and low-temperature *π* and low-pass RC filters at 0.35 K to minimise the electrical noise on the sample. Electrical resistance was measured in standard four-probe configuration using DC delta mode with bias current 1 *μ*A to 10 *μ*A determined by the sample resistance and signal to noise ratio. Magnetic fields with desired field orientations were applied by the vector magnet at one cooling without altering sample position.

### Raman spectroscopy

Raman spectra were obtained using a confocal microscopy set-up with laser beam size of ~ 1 *μ*m and laser power of ~ 1 mW. A HeNe laser (632.8 nm) was used to excite IrTe_2_ flakes in an optical cryostat at normal incidence. The Raman signal was collected in the backscattering configuration and analysed using a monochromator equipped with a liquid nitrogen-cooled silicon CCD. Two linear polarizers in the parallel configuration were placed immediately after the laser and before the monochromator to define the polarization of incident and scattered light, respectively. The crystal orientation relative to the polarisation of the incident light was controlled using a half waveplate between a beam splitter and IrTe_2_ flakes. The sample position was precisely controlled using a piezo stage.

### Scanning tunnelling microscopy and spectroscopy

For scanning tunnelling microscopy (STM) and spectroscopy (STS) measurements, IrTe_2_ nanoflakes were exfoliated in a glove box (H_2_O < 0.1 ppm, O_2_ < 0.1 ppm) filled with Ar gas, and transferred onto graphene, grown epitaxially on a 4H-SiC(0001) substrate. The samples were then transferred to a ultrahigh vacuum chamber (*P* ≤ 1 × 10^−10^ Torr) for STM/STS measurements without any exposure to air to ensure clean surfaces of IrTe_2_ nanoflakes. STM images were typically obtained using a bias voltage *V*_b_ = − 2.5 V and a tunnelling current *I*_t_ = 20 pA for large-scale imaging; *V*_b_ = 15 mV and *I*_t_ = 1 nA for charge-ordered stripe phases; *V*_b_ = 5 mV and *I*_t_ = 2 nA for atomically-resolved images. For STS, we used the lock-in technique with a bias modulation of 7 mV_rms_.

### Density functional theory calculations

Density functional theory (DFT) calculations for electronic structures and the electron-phonon coupling (EPC) were performed by the Quantum Espresso package implementing the pseudo-potential band method and the density functional perturbation theory^52,53^. We utilised Perdew-Burke-Ernzerhof sol (PBEsol, revised PBE for solid)^54^ as an exchange-correlation functional and included the spin-orbit coupling (SOC). The dynamical matrices were calculated using 2 × 2 × 2 *q*-mesh and 16 × 10 × 4 *k*-mesh with 40 Ry energy cutoff. We applied the various in-plane tensile strains in the range of Δ*a*/*a* ~ 2.1–3.1% and the fixed compressive strain of Δ*b*/*b* ~ 0.65% to model the experimental situations (Supplementary Note 6). Atomic positions were optimised in each case.

## Supplementary information

Supplementary Information

Peer Review File

## Data Availability

The data that support the findings of this study are available from the corresponding authors on request.
