# Supplementary material for: Superconductivity emerging from a stripe charge order in IrTe2 nanoflakes
Source: Nat Commun. 2021 May 26;12:3157. doi: 10.1038/s41467-021-23310-w (PMC8154908; doi:10.1038/s41467-021-23310-w)
Supplement: Supplementary file 1 — Supplementary Information [file 41467_2021_23310_MOESM1_ESM.pdf]

Supplementary Information: Superconductivity emerging from a stripe charge order in $IrTe_2$ nanoflakes

Sungyu Park,[1,*] So Young Kim,[1,2,*] Hyoung Kug Kim,[2] Min Jeong Kim,[1,3] Taeho Kim,[1,3] Hoon Kim,[1,2] Gyu Seung Choi,[1,2] C. J. Won,[4,5] Sooran Kim,[6] Kyoo Kim,[7] Evgeny F. Talantsev,[8,9] Kenji Watanabe,[10] Takashi Taniguchi,[11] Sang-Wook Cheong,[4,5,12] B. J. Kim,[1,2] H. W. Yeom,[1,2] Jonghwan Kim,[1,2,3,†] Tae-Hwan Kim,[2,5,13,‡] and Jun Sung Kim[1,2,§]

[1]*Center for Artificial Low Dimensional Electronic Systems, Institute for Basic Science, Pohang 37673, Korea*

[2]*Department of Physics, Pohang University of Science and Technology, Pohang 37673, Korea*

[3]*Department of Materials Science and Engineering, Pohang University of Science and Technology, Pohang 37673, Korea*

[4]*Laboratory for Pohang Emergent Materials, Pohang Accelerator Laboratory, Pohang 37673, Korea*

[5]*Max Planck POSTECH/Korea research Initiative, POSTECH, Pohang 37673, Korea*

[6]*Department of Physics Education, Kyungpook National University, Daegu, 41566, Korea*

[7]*Korea Atomic Energy Research Institute (KAERI), 111 Daedeok-daero 989 Beon-Gil, Yuseong-gu, Daejeon, 34057, Korea*

[8]*M.N. Mikheev Institute of Metal Physics, Ural Branch, Russian Academy of Sciences, 18, S. Kovalevskoy St., Ekaterinburg, 620108, Russia*

[9]*NANOTECH Centre, Ural Federal University, 19 Mira St., Ekaterinburg, 620002, Russia*

[10]*Research Center for Functional Materials, National Institute for Materials Science, 1-1 Namiki, Tsukuba 305-0044, Japan*

[11]*International Center for Materials Nanoarchitectonics, National Institute for Materials Science, 1-1 Namiki, Tsukuba 305-0044, Japan*

[12] *Rutgers Center for Emergent Materials and Department of Physics and Astronomy, Rutgers University, Piscataway, New Jersey 08854, USA*

[13] *Asia Pacific Center for Theoretical Physics (APCTP), Pohang 37673, Korea*

(Dated: May 4, 2021)

---

* equal contribution

† jonghwankim@postech.ac.kr

‡ taehwan@postech.ac.kr

§ js.kim@postech.ac.kr

Supplementary Note 1: Exfoliation and stability

The thickness of exfoliated $IrTe_2$ crystals was estimated by atomic force microscopy (AFM). The optical and AFM images for typical $IrTe_2$ nanoflakes on top of a SiO_2/Si substrate are shown in Supplementary Fig. 1. The obtained flakes are typically several μm^2 in area and 4–160 nm in thickness, as indicated by a line profile in the insets of Supplementary Fig. 1. We found that the optical contrast, AFM thickness, and Raman spectra of $IrTe_2$ nanoflakes are nearly unchanged even after a month in ambient condition, as shown in Supplementary Fig. 1. These results confirm that nanometer-thick and stable $IrTe_2$ flakes can be successfully isolated from a bulk crystal.

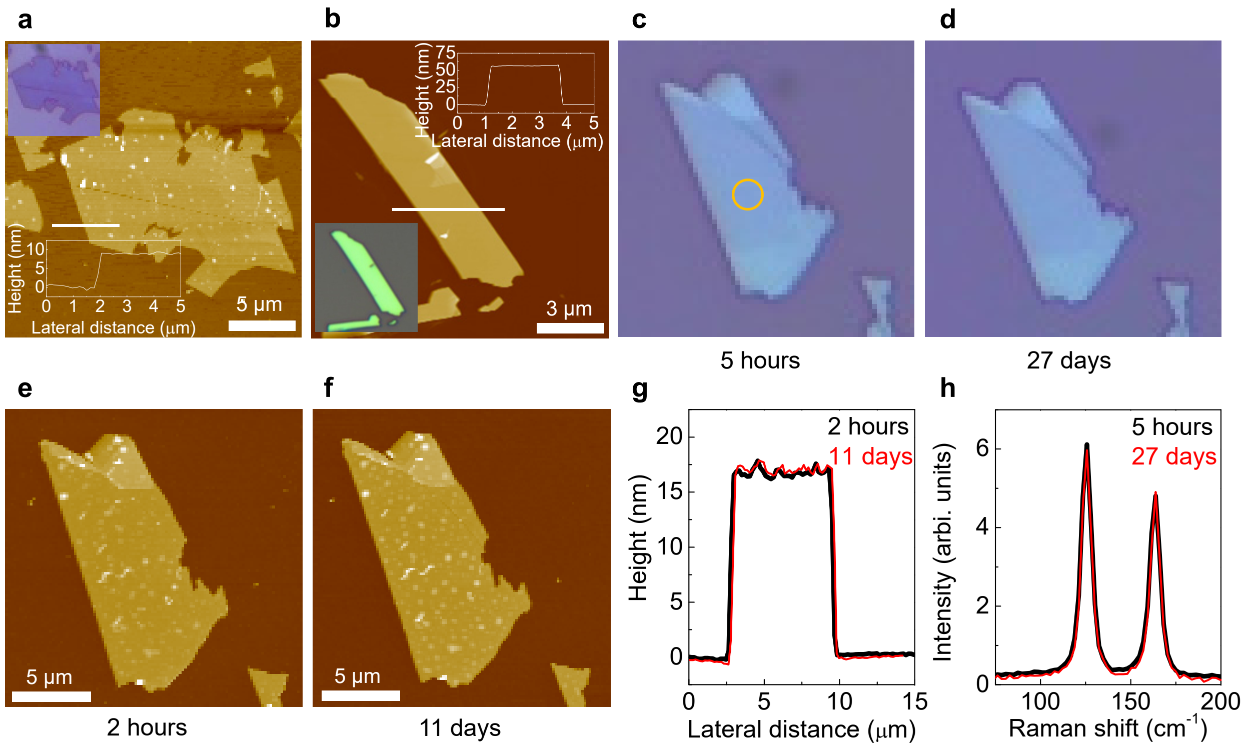

Supplementary Fig. 1. **Atomic force microscopy and optical images of $IrTe_2$ nanoflake.** **a**,**b**, AFM images of (**a**) 9-nm-thick and (**b**) 56-nm-thick $IrTe_2$ nanoflakes. The insets show optical images and AFM line profiles of the nanoflakes. **c**,**d**, Optical images of 16-nm-thick nanoflake of $IrTe_2$ after exfoliated in the air. **e**–**g**, AFM image and line profiles obtained right after exfoliation and 11 days later. **h**, Raman spectra examined right after exfoliation and 27 days later at the indicated points in **c**.

Supplementary Note 2: Raman spectroscopy

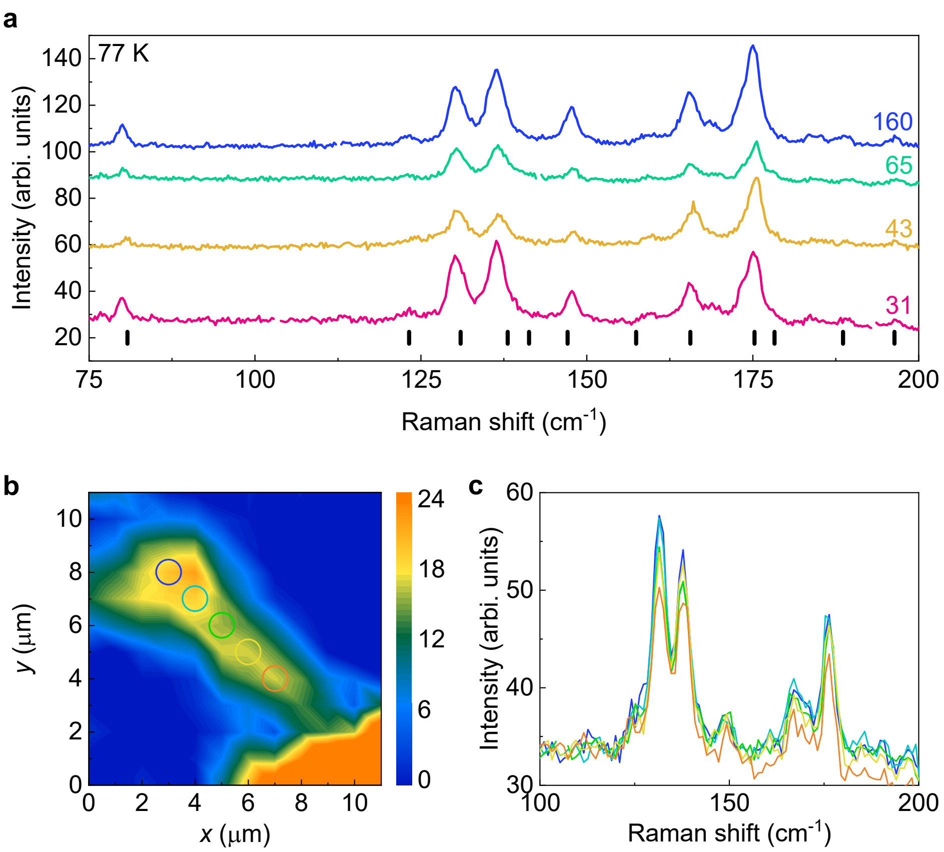

Supplementary Fig. 2. **Spatial Raman intensity map of 43-nm-thick $IrTe_2$ nanoflake.** **a**, Raman spectra of nanoflakes with d = 31, 43, 65, and 160 nm at 77 K below T_s. Observed Raman modes are consistent with those of bulk $IrTe_2$ as indicated by the black vertical bars at the bottom [1]. **b**, Spatially resolved Raman intensity map of low temperature mode at 129 cm^{-1}. **c**, Raman spectra at the indicated points in **b**.

The stripe charge order in $IrTe_2$ nanoflakes is characterised using Raman spectroscopy below T_s. For several nanoflakes with different thicknesses ranging from 31 nm to 160 nm, we found multiple peak splitting in the Raman spectra taken at 77 K well below T_s. These splittings are attributed to the lowered symmetry and the emergence of a super unit cell during the structural transition, similar to the case of bulk $IrTe_2$ [1–3]. Such a stripe ordering occurs in the entire area of the nanoflake as found *e.g.* in the Raman intensity map taken on the 43-nm-thick flake. We collected the intensity of the Raman mode at 129 cm^{-1}, the fingerprint of the stripe phase, throughout the flake with a step size of 1 μm, as shown in

Supplementary Fig. 2b. All the Raman spectra, obtained at different positions (circles in Supplementary Fig. 2b), are nearly the same and show clear peak splittings (Supplementary Fig. 2c). The slight inhomogeneity of the intensity across the flake can be explained by domain formation with different stripe orientations, because the Raman intensity depends on orientations of the stripe patterns and the linear polarisation of the incident laser beam. This is consistent with the scanning tunnelling microscopy (STM) results showing domain formation of three energetically equivalent stripe orientations (Supplementary Fig. 5 and Fig. 2d).

The dominant stripe phase formation near the superconducting transition at T_c is further confirmed by Raman spectroscopy on fifteen nanoflakes with different thicknesses, ranging from 10 nm to 174 nm (Supplementary Fig. 3c). For all the flakes cooled down to 4 K (Supplementary Fig. 3a), we observed multiple peak splitting of Raman modes without any signature of the Raman modes for the high-temperature normal phase. Thus we conclude that the region of the stripe charge order phase encompasses the whole superconducting dome (Supplementary Fig. 3b) in the thickness-dependent phase diagram, and the stripe-charge-order stable at 4 K, just above $T_c \sim 2$ K, serves as the parent state for the superconducting state. These results are consistent with the significantly enhanced out-of-plane superconducting coherence length found in Fig. 3.

Supplementary Note 3: Scanning tunnelling microscopy/spectroscopy

To investigate the structural and electronic properties of $IrTe_2$ nanoflakes at room temperature, we performed scanning tunnelling microscopy and spectroscopy (STM/STS). Supplementary Fig. 4 shows STM/STS results for a typical $IrTe_2$ nanoflake with thickness of 120 nm. The STM image exhibits the whole flake with the clean and flat surface, which has the well-defined edges along three crystalline directions due to its hexagonal symmetry (Supplementary Fig. 4a). The atom-resolved STM image and its fast Fourier transform confirm that the topmost hexagonal Te atoms show no charge-ordered phase (Supplementary Fig. 4b). The unit cell, Te, and Ir atoms are indicated by black parallelogram, grey and green balls, respectively. Supplementary Fig. 4c describes the local density of states (LDOS) of the $IrTe_2$ nanoflake, showing its metallic behaviour. Comparing with bulk $IrTe_2$, we can conclude that the intrinsic characteristics of the structural and electronic properties on thin

nanoflakes remain the same at room temperature [4].

At low temperature (T_{STM} = 85 K), we found three energetically equivalent charge-ordered domains on $IrTe_2$ nanoflake (Supplementary Fig. 5). We have rigorously mapped out the nanoflake by taking more than a hundred of STM images resolving charge-ordered phases along all domain boundaries. We could find only three stripe ordered domains as expected from three-fold rotational symmetry, but no hexagonal ordered phases which can be superconducting at lower temperatures. In bulk $IrTe_2$ crystals, superconducting hexagonal

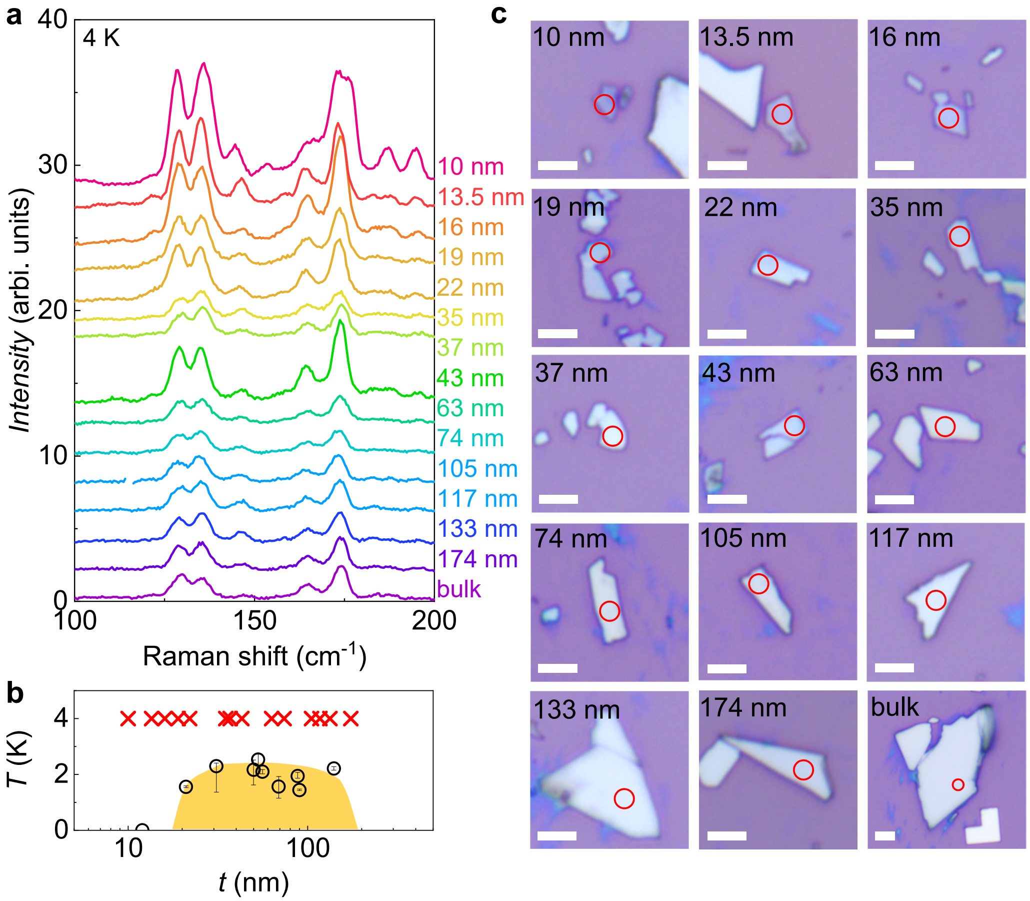

Supplementary Fig. 3. **Raman spectra of $IrTe_2$ nanoflakes at 4 K.** **a**, Raman spectra of nanoflakes with $10 \leq d \leq 174$ nm and bulk at 4 K. **b**, Thickness-dependent phase diagram with a superconducting dome (Black). Red crosses indicate the thickness and temperature points at which the stripe-charge-order formation is confirmed by Raman spectroscopy. **c**, Optical images of nanoflakes used for Raman spectroscopy (scale bar, 3 μm). The positions where the laser was focused are indicated by red circles.

ordered phases appear at the specific locations where three striped phases meet [5]. It is noteworthy that bulk crystals always have non-negligible portion of hexagonal domains upon rapid thermal quenching. Thus, we carefully examined the existence of the hexagonal phase after rapid thermal quenching (1 K/sec) especially at A and B indicated in Supplementary Fig. 5a. As shown in Supplementary Figs. 5b–e, we confirmed that no hexagonal phase exists even though the three different stripe phases meet. The same conclusion can be drawn for even thinner flakes as shown in Supplementary Figs. 6a–c. As shown in atomically resolved STM images (Supplementary Figs. 6d–f), we observed that these thin nanoflakes, with thicknesses ranging from 11 to 20 nm, exhibit the dominant stripe ordering with period of $5a_0$, which is different from that ($8a_0$) of bulk $IrTe_2$ at the same temperature [6].

Supplementary Note 4: Transport properties

The thermal cycling effect on the $IrTe_2$ nanoflakes was investigated by measuring the in-plane resistivity. Supplementary Fig. 7 shows the temperature-dependent resistivity $\rho(T)$ curves, normalised by its room temperature value $\rho(300\text{ K})$, for the 56 nm-thick nanoflake, which were taken at different thermal cycles with different temperature ramping rates of

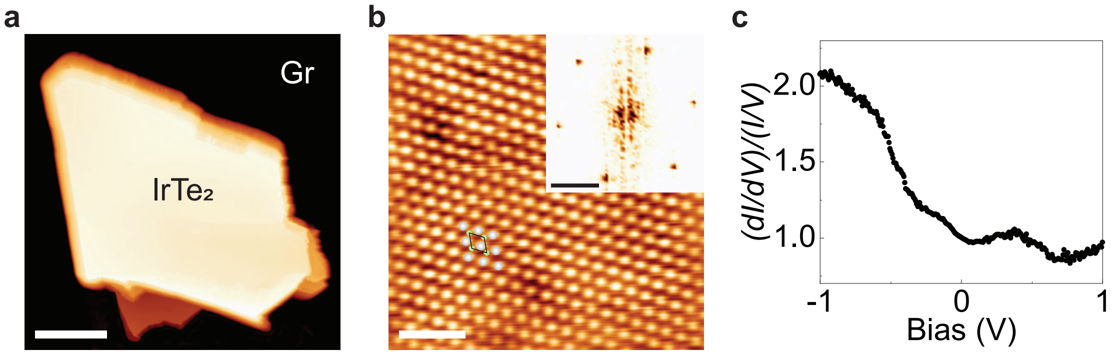

Supplementary Fig. 4. **Scanning tunnelling microscopy and spectroscopy (STM/STS) of a $IrTe_2$ nanoflake at room temperature.** **a**, STM image of a 120-nm-thick $IrTe_2$ flake on a graphene substrate (scale bar, 500 nm; $V_s = 1.0$ V; $I_t = 0.02$ nA). **b**, Atom-resolved STM image of **a** exhibiting the hexagonal structure (scale bar, 2 nm; $V_s = -4$ mV, $I_t = 5.0$ nA). Grey and green balls represent Te and Ir atoms on **b**. The inset of **b** is the fast Fourier transformation of **b** (scale bar, 2 nm^{-1}). **c**, The normalised STS spectra $(\frac{dI}{dV})/(\frac{I}{V})$ obtained on top of the nanoflake.

0.3–4 K/min. The weak but clear resistive anomalies, due to the stripe charge ordering, were repeatedly observed at $T_{s,\mathrm{dn}}$ = 60–80 K for cooling (Fig. 7a) and at $T_{s,\mathrm{up}}$ = 180–210 K for warming (Supplementary Fig. 7b). The transition temperatures $T_{s,\mathrm{dn}}$ and $T_{s,\mathrm{up}}$ vary at different cooling and warming runs, consistent with previous reports [7]. The superconducting transitions, however, are almost identical at different cooling runs. As shown in Supplementary Fig. 7c, the superconducting transition temperature T_c = 2.08 K and the width ΔT_c = 0.05–0.07 K remain the same on several thermal cycles, indicating stable superconducting phase in $IrTe_2$ nanoflakes.

Upper critical field B_{c2} of $IrTe_2$ nanoflakes was determined by magnetoresistance (MR) below T_c. The MR curves of four representative nanoflakes with d = 21, 56, 90, and 140 nm, are shown in Supplementary Figs. 8a–h for $B \| ab$ and $B \| c$. Most of the samples exhibit a relatively sharp transition, but in some cases with *e.g.* d = 140 nm, intermediate steps in $\rho(B)$ during the transition were observed for $B \| c$. However, such multiple steps in MR are suppressed at higher temperatures, close to T_c (Supplementary Figs. 8e–h), and also for $B \| ab$ (Supplementary Figs. 8a–d). These observations indicate that the presence of macroscopic

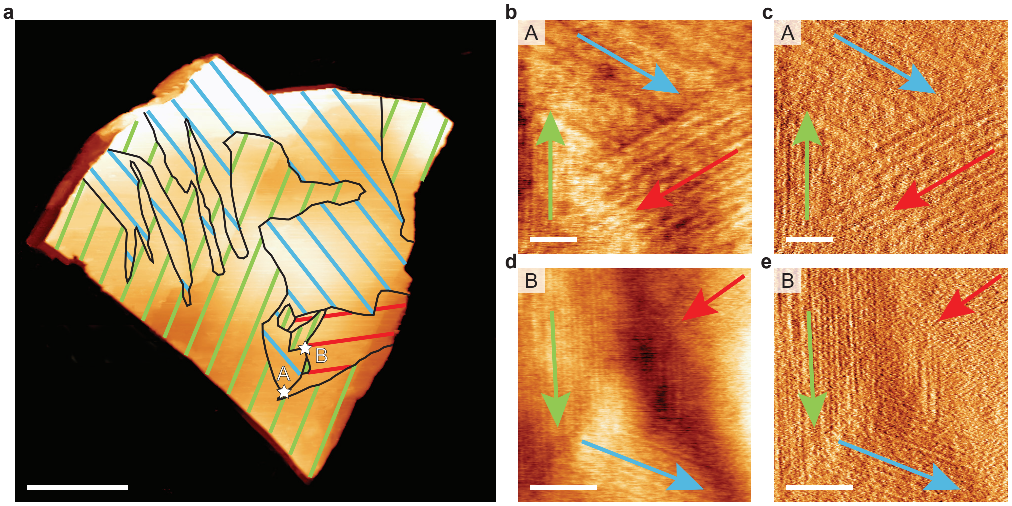

Supplementary Fig. 5. **Stripe domains and their boundaries at 85 K.** **a**, 80-nm-thick $IrTe_2$ flake on a graphene substrate (scale bar, 500 nm; V_s = −1.0 V; I_t = 0.02 nA). Coloured lines represent the stripe directions of charge-ordered domains. **b**–**e**, STM topography images $Z(x, y)$ (**b**,**d**) and their derivative $\frac{dZ}{dx}$ (**c**,**e**) at the A and B sites in **a**. Over the entire surface of the nanoflake, only three equivalent stripe domains coexist, showing no hexagonal phase that becomes superconducting below the transition temperature. Scale bar, (**b**,**c**) 10 nm and (**d**,**e**) 20 nm.

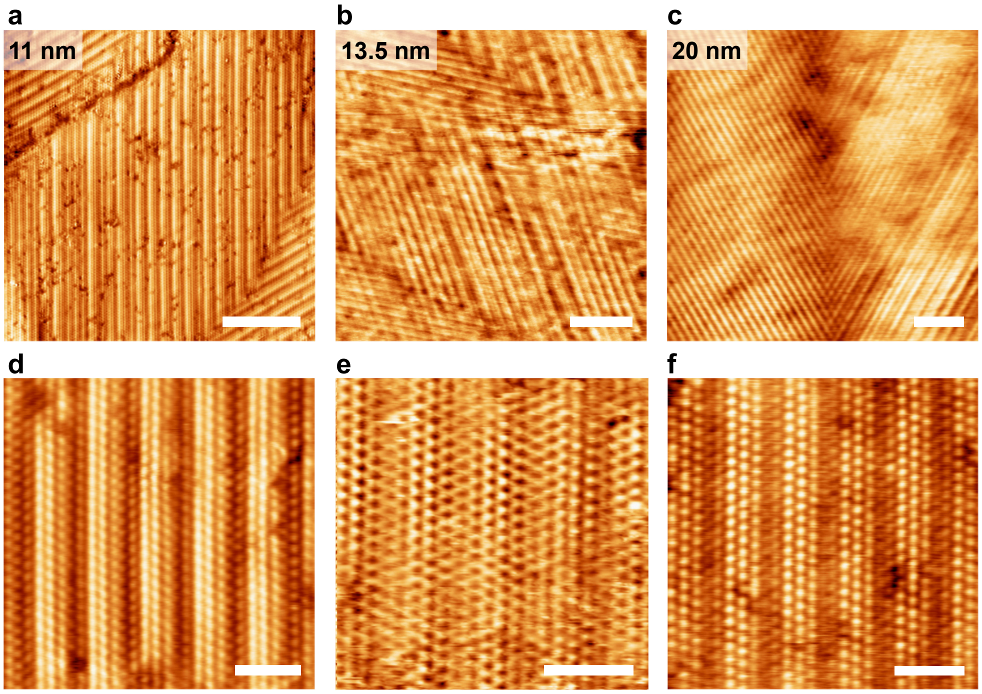

Supplementary Fig. 6. **Stripe charge order formation on ultra-thin $IrTe_2$ nanoflakes at 85 K. a–c**, STM topographic images of nanoflakes with thicknesses of (**a**) 11 nm, (**b**) 13.5 nm, and (**c**) 20 nm. All nanoflakes exhibit only the stripe charge ordering without hexagonal phases. **d–f**, Atom-resolved STM images of **a–c** representing the dominant stripe charge ordering with period of $5a_0$. Note that **c** and **f** are the same as Fig. 2e and 2f, respectively. Scale bar, (**a–c**) 10 nm and (**d–f**) 2 nm.

domains with different T_c or H_{c2} is unlikely to be the origin of the observed multi-steps in MR. Instead, such a behaviour can be induced by the weak links and the associated vortex dynamics in 2D superconductors [8–10]. As found in the STM results (Supplementary Fig. 6 and Fig. 2), the stripe-charge-ordered domains with different orientations are formed in $IrTe_2$ flake. The resulting domain boundaries, known to have a high resistance [11], may serve as weak links, which are more fragile to external currents or magnetic fields and produce the multi-steps, particularly for $B\|c$. The $B_{c2}(T)$ curves, determined using three different criteria, 10, 50 and 90% of the resistive transition, are consistent with each others (Supplementary Figs. 8i–l), particularly for $B\|ab$. The resulting $B_{c2}(T)$ curves exhibit distinct temperature dependence for $B\|ab$ and $B\|c$. $B_{c2}^{ab}(T)$ for $B\|ab$ follows the typical behaviour of two dimensional superconductors, well described by $B_{c2}^{ab}(T) \propto (1 - T/T_c)^{1/2}$.

This is distinct from the almost T-linear dependence of $B^c_{c2}(T)$ for $B \| c$ [12, 13]. We thus conclude that 2D superconductivity is realised in $IrTe_2$ nanoflakes.

The field orientation dependence of $B_{c2}(\theta)$ further supports the same conclusion. The MR curves of four representative samples with d = 21, 56, 90, and 140 nm, are shown in Supplementary Figs. 9a–d, with different field angles (θ) against the in-plane. The corresponding $B_{c2}(\theta)$ data as a function of field angle, determined by 50% resistive transition, commonly exhibit strong anisotropy (Supplementary Figs. 9e–h) and, more importantly, a clear cusp at $\theta = 0$ for all the nanoflakes (Supplementary Figs. 9i–l). We employed the 2D Tinkham model [14] and 3D Ginzburg-Landau (GL) model [12] to fit the $B_{c2}(\theta)$ data taken in the whole angle range (dashed lines) or within $\pm 1^\circ$ of the in-plane magnetic field (solid lines) as shown in Supplementary Figs. 9e-l. Because the 3D GL model describes the angle dependency of $B_{c2}(\theta)$ of a single anisotropic 3D Fermi surface, it cannot reproduce $B_{c2}(\theta)$ for multiband superconductors for the whole range of angle. However, in the narrow angle region near the in-plane field, *e.g.* $|\theta| \leq 1^\circ$, these models can reproduce the $B_{c2}(\theta)$ data well, depending on the dimensionality of superconductivity. Particularly, a cusp in $B_{c2}(\theta)$

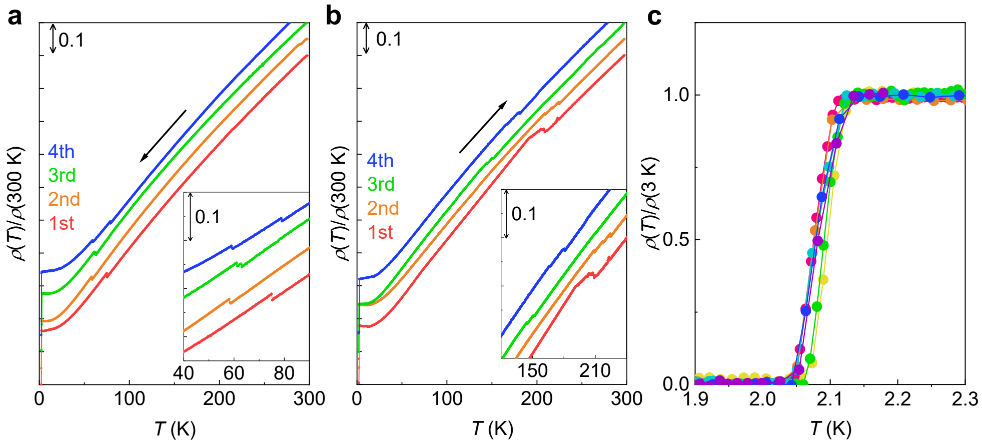

Supplementary Fig. 7. **Temperature dependence of the resistance at several thermal cycles.** **a**,**b**, Temperature dependence of the normalised resistivity $\rho(T)/\rho(300\text{ K})$ curves at four thermal cycles in the temperature range from 300 to 0.3 K when cooling down and warming up, respectively. The insets show magnification of the regions where the resistance jumps due to structure transition. For clarity, $\rho(T)/\rho(300\text{ K})$ curves are offset vertically. **c**, Temperature dependence of the normalised resistivity $\rho(T)/\rho(3\text{ K})$ curves near superconducting transition at seven thermal cycles.

at $\theta = 0°$ is expected only in the 2D Tinkham model, but not in the anisotropic 3D GL model. We found that for $d < 100$ nm, the angle dependence of $B_{c2}(\theta)$ shows a clear cusp at $\theta = 0°$, confirming the 2D superconductivity of $IrTe_2$ nanoflakes. For $d = 140$ nm, the anisotropy of $B_{c2}(\theta)$ is significantly suppressed to only a few % within $|\theta| < 1°$, in contrast to the case of the thinner samples, *e.g.* $\sim 26\%$ for the 21 nm-thick flake, which makes the

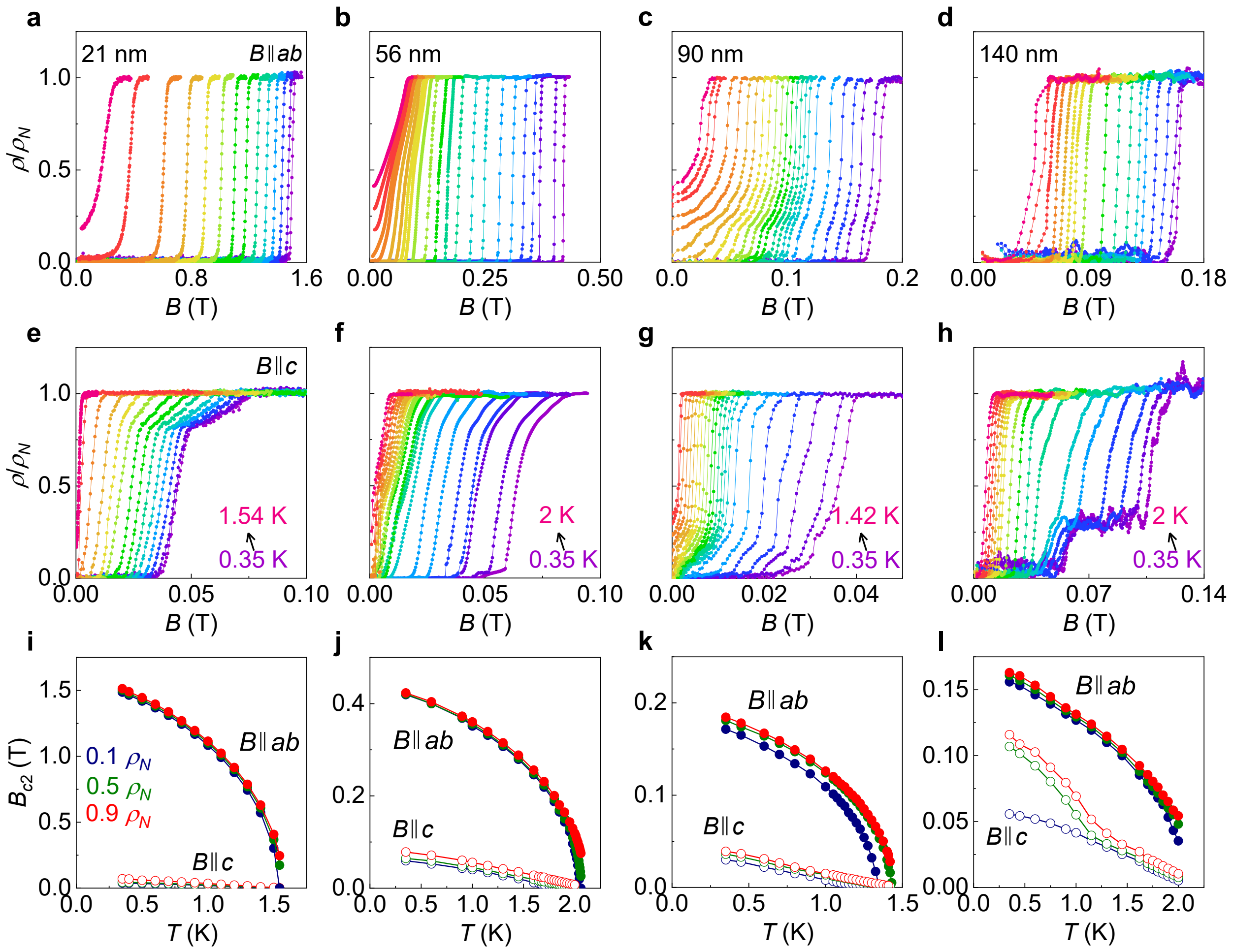

Supplementary Fig. 8. **Temperature dependent B_{c2} curves determined by different criteria. a–d**, Magnetoresistance for in-plane magnetic fields at different temperatures from 0.35 K to T_c of $IrTe_2$ nanoflakes with thickness $d = 21$ (**a**), 56 (**b**), 90 (**c**), and 140 nm (**d**). **e–h**, Magnetoresistance for out-of-plane magnetic fields at different temperatures for the nanoflakes. **i–l**, Corresponding in-plane (solid) and out-of-plane (open) B_{c2} depending on temperature with different B_{c2} determinations, 10 (blue), 50 (green), and 90% (red) of the resistive transition for the nanoflakes.

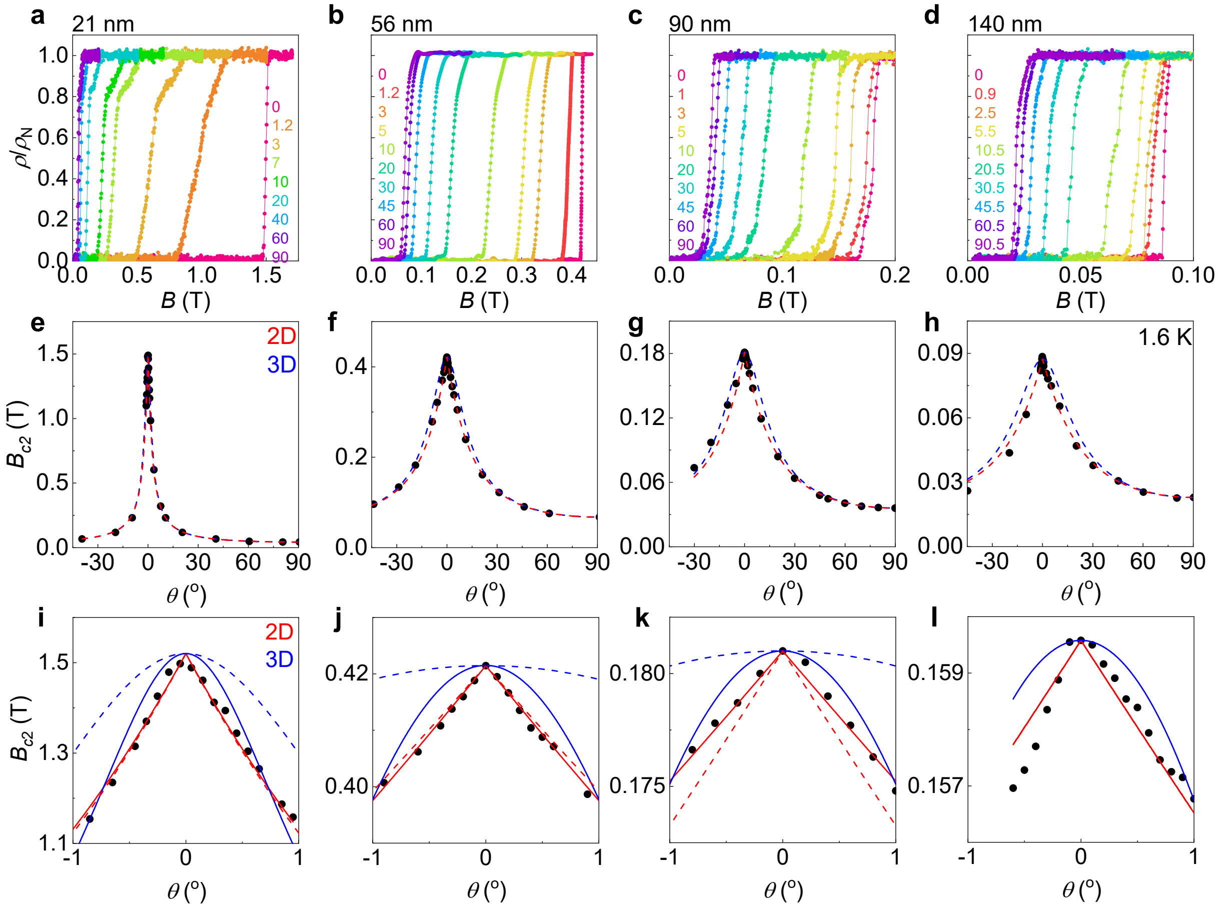

Supplementary Fig. 9. **Angle dependent B_{c2} curves determined by different criteria.** **a–d**, Magnetic field dependence of the normalised resistivity curves with various angles in $IrTe_2$ nanoflakes with thickness of 21 (**a**), 56 (**b**), 90 (**c**), and 140 nm (**d**). **e–h**, Corresponding B_{c2} depending on angles, determined by 50% resistive transition for the nanoflakes. The measurements were done at $T = 0.35$ K, otherwise noted. Red and blue dashed lines are the fits of the whole angle dependent data to the 2D Tinkham and 3D Ginzburg-Landau models, respectively. **i–l**, Magnified angle dependent B_{c2} near the in-plane magnetic field for $|\theta| \leq 1°$. Red and blue solid lines are the fits of $B_{c2}(\theta)$ data to the 2D Tinkham and 3D Ginzburg-Landau models, respectively. The dashed lines are the fit of the whole angle dependent data as shown in **e–h**

.

precise determination of $B_{c2}(\theta)$ difficult. Nevertheless, the observed $B_{c2}(\theta)$ data show a clear difference from the anisotropic 3D Ginzburg-Landau model, but agree reasonably well with the 2D Tinkham model (Supplementary Fig. 9l and Fig. 3c). These results indicate that

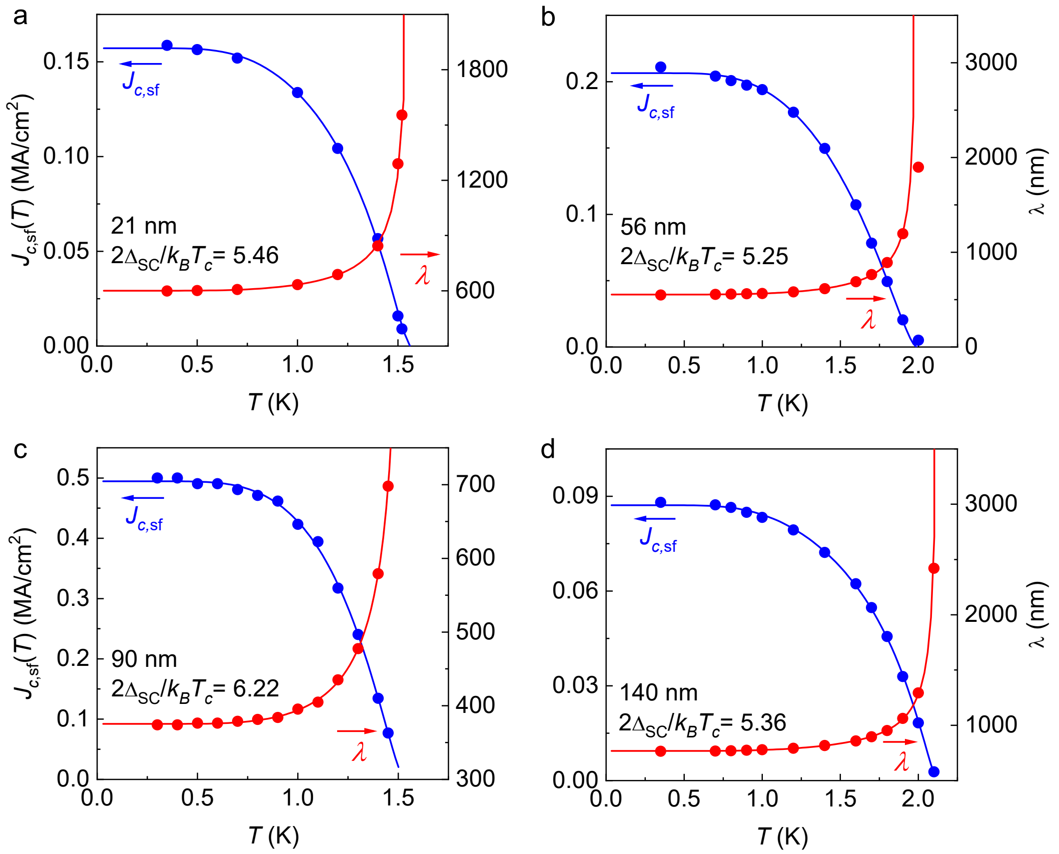

Supplementary Fig. 10. **Temperature dependent $J_{c,sf}$ curves.** Temperature dependence of $J_{c,sf}(T)$ and $\lambda(T)$ data together with fits for $IrTe_2$ nanoflakes with thickness of 21 (**a**), 56 (**b**), 90 (**c**), and 140 nm (**d**).

the 2D superconductivity is realised even for relatively thick $IrTe_2$ flakes.

Supplementary Note 5: Superconducting gap estimation

Superconducting gap of thin films or nanoflakes can be extracted from the temperature dependent self-field critical current [15–17]. When the thickness d of the superconducting system is much smaller than the penetration depth $\lambda(0)$ ($d \ll \lambda$), superconductivity is destroyed by excessive self-field, induced by the supercurrent. In this case, the temperature dependent self-field critical current density, $J_{c,sf}(T)$, is closely related with London penetration depth, $\lambda(T)$, which is determined by the size and the type of superconducting gap $\Delta_{SC}(T)$ such as single or multiple gaps and s- or d-wave gaps. Talantsev *et al.* successfully demonstrated that $\lambda(T)$ and $\Delta_{SC}(0)$, extracted from $J_{c,sf}(T)$ in superconducting thin films,

are in excellent agreement with those measured using other techniques for a wide range of superconducting thin films of metals, nitrides, oxides, cuprates, pnictides, borocarbides, MgB_2, and heavy fermions [15–17], which is recently extended to various superconducting nanoflakes including $NbSe_2$ [16], FeSe on $SrTiO_3$ [16], $PdTe_2$ on $SrTiO_3$ [18], and ionic gated MoS_2 [19].

The temperature dependent $J_{c,\mathrm{sf}}(T)$ data for representative $IrTe_2$ nanoflakes with $d = 21$, 56, 90, and 140 nm are shown in Supplementary Fig. 10. The penetration depth for doped $IrTe_2$ is found to be $\sim$ 150 nm [20], much larger than the thickness of the nanoflakes. This ensures to apply the self-field model to the measured $J_{c,\mathrm{sf}}(T)$ data. In all cases, $J_{c,\mathrm{sf}}(T)$ and the corresponding $\lambda(T)$ data are in good agreement with the fits to the model, assuming a single s-wave gap with BCS temperature dependence [21]. From the extracted $\Delta_{\mathrm{SC}}(0)$ and the measured T_c, we obtained the gap ratio $2\Delta_{\mathrm{SC}}/k_B T_c \sim 5.6$, which is much larger than the BCS value of $2\Delta_{\mathrm{SC}}/k_B T_c = 3.53$. These results show that superconductivity in $IrTe_2$ nanoflakes is in the strong coupling regime.

Supplementary Note 6: Density functional theory calculations

When a nanoflake is exfoliated and anchored by metal electrodes on top of a substrate, the in-plane strain is expected to be applied at low temperatures because of the different thermal contraction between the nanoflake and the substrate (Supplementary Fig. 11a). For $IrTe_2$ nanoflakes in stripe charged phase, the thermal expansion coefficients are $\alpha \sim +110\times10^{-6}$ K^{-1} along the a-axis and $\sim -21\times10^{-6}$ K^{-1} along the b-axis [22], much larger in magnitude than those of Si ($\alpha \sim +2.56\times10^{-6}$ K^{-1}) [23] and other 2D materials *e.g.* MoS_2 ($\alpha \sim +7\times10^{-6}$K^{-1}) [24]. Using these thermal expansion coefficients we estimate the substrate-induced tensile strain up to $\Delta a/a \sim 3\%$ and $\Delta b/b \sim -0.7\%$ at 20 K. In the realistic case, there are domains of stripe order with different orientations in $IrTe_2$ nanoflake, which releases the substrate-induced strain. The resulting strain would be moderate, 0.1–0.3% [25], but has a strong impact on the stability of the stripe-charge-order phases as found in a recent study [26] . The strain-induced changes of the periodicity and also the Ir-Ir dimer density significantly modify the charge transfer between Ir and Te states and thus the electronic structures.

To investigate the effect of the substrate-induced strain, we performed the density func-

tional theory calculations for estimating electron-phonon coupling and T_c. Supplementary Fig. 11 shows the phonon dispersion curves and electron-phonon coupling (EPC) of the stripe phase without and with the 3.0% in-plane tensile strain. When the in-plane strain becomes larger than a critical value of 3.1%, we observed the imaginary phonon frequency near the Γ point, indicating the structural instability. However, this critical strain, estimated from calculations, is far larger than the experimental one, $\sim 0.1\%$ [26]. The observed extreme sensitivity of the stripe-charge-ordered phases to the strain implies that the $5a_0$ stripe phase is much closer to the structural instability than expected in DFT-based calculations. Thus the calculations would capture the qualitative nature of phonon softening due to the

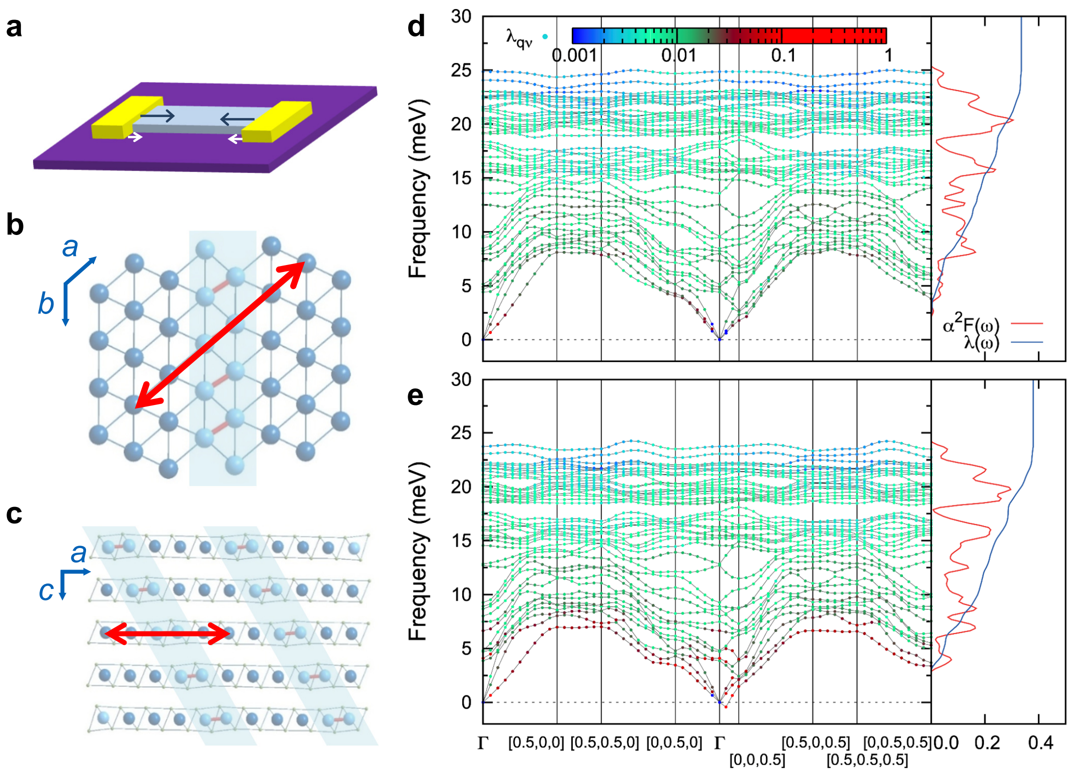

Supplementary Fig. 11. **Strain effect on $IrTe_2$.** **a**, Thermal contraction of $IrTe_2$ nanoflake on Si substrate. Black and white arrows show thermal contraction of $IrTe_2$ and Si, respectively. **b**,**c**, Strain direction for the maximum difference of thermal contraction between nanoflake and Si substrate. The length of red arrows means $5a_0$. **d**,**e**, Phonon dispersion curves with electron-phonon coupling constant λ_{qv}, the Eliashberg function $\alpha^2F(\omega)$, and $\lambda(\omega)$ of stripe phase structure (**d**) without the in-plane strain and (**e**) with the 3.0% in-plane strain. The colour in the phonon dispersion curves indicates the magnitude of λ_{qv}.

in-plane strain (%)	Ir-Ir dimer distance (Å)	$N(E_F)$ (states/e.V./f.u.)	$\omega_{\log}$ (K)	$\lambda_{\rm ep}$	T_c (K)
0	3.099	1.069	139.44	0.3351	0.048
2.1	3.208	1.113	134.41	0.3464	0.067
2.6	3.238	1.139	129.32	0.3624	0.101
3.0	3.259	1.159	124.69	0.3793	0.148

Supplementary Table. I. Superconducting parameters and the Ir-Ir dimer distance in the stripe order depending on the in-plane strain. T_c was estimated by using μ^* of 0.13.

structural instability and the resulting enhancement of the electron-phonon coupling with increase of the strain.

The magnitude of electron-phonon coupling constant $\lambda_{\rm qv}$ is indicated by the colour in the dispersion curves. We computed the superconducting parameters, such as the logarithmic averaged phonon frequency $\omega_{\log}$, the electron-phonon coupling (EPC) constant $\lambda_{\rm ep}$, and superconducting temperature T_c using the Eliashberg EPC and Allen-Dynes formula [27, 28],

$$\omega_{\log} = \exp\left[\frac{2}{\lambda_{\rm ep}}\int\frac{d\omega}{\omega}\alpha^2F(\omega)\log\omega\right], \tag{1}$$

$$\lambda_{\rm ep} = 2\int\frac{d\omega}{\omega}\alpha^2F(\omega), \tag{2}$$

$$T_c = \frac{\omega_{\log}}{1.20}\exp\left[\frac{-1.04(1+\lambda_{\rm ep})}{\lambda_{\rm ep}(1-0.62\mu^*)-\mu^*}\right], \tag{3}$$

where $\alpha^2F(\omega)$ is the Eliashberg function and μ^* is the effective Coulomb repulsion parameter. The superconducting parameters depending on the in-plane strain are summarised in Table I. We have found that the enhanced EPC and T_c with increasing in-plane strain from 0 to 3.0%. With increasing in-plane strain, the overall phonon bands become softer resulting in increasing $\lambda_{\rm ep}$, which follows the equation $\lambda_{\rm ep} \propto \frac{1}{\langle\omega^2\rangle}$ where $\langle\omega^2\rangle$ is the averaged phonon-frequency square [29]. Especially, $\alpha^2F(\omega)$ with 3.0% strain shows the two additional peaks in the low frequency range of 4 meV and 7 meV, which contribute the enhanced $\lambda_{\rm ep}$. In contrast, assuming elongation of the c-axis lattice parameter up to $\Delta c/c \sim 9\%$, two orders of magnitude larger than typically found in other nanoflakes [30, 31], we found much smaller change in $\lambda_{\rm ep}$ and T_c. Considering the calculated T_c smaller than observed in experiments, we cannot rule out the possibility that superconductivity in $IrTe_2$ is not fully captured by

the DFT-based calculations. Nevertheless, the clear trend of λ_{ep} and T_c suggests that the tensile strain, rather than interlayer elongation, may play an important role for suppression of the stripe order and appearance of superconductivity in $IrTe_2$ nanoflakes.

---

[1] Glamazda, A., Choi, K.-Y., Lemmens, P., Yang, J. J. & Cheong, S.-W. Proximity to a commensurate charge modulation in $IrTe_{2-x}Se_x$ ($x = 0$ and 0.45) revealed by Raman spectroscopy. *New J. Phys.* **16**, 93061 (2014).

[2] Cao, H. *et al.* Origin of the phase transition in $IrTe_2$: Structural modulation and local bonding instability. *Phys. Rev. B* **88**, 115122 (2013).

[3] Pascut, G. L. *et al.* Dimerization-Induced Cross-Layer Quasi-Two-Dimensionality in Metallic $IrTe_2$. *Phys. Rev. Lett.* **112**, 86402 (2014).

[4] Kim, H. S., Kim, T.-H., Yang, J., Cheong, S.-W. & Yeom, H. W. Structural versus electronic distortions in $IrTe_2$ with broken symmetry. *Phys. Rev. B* **90**, 201103 (2014).

[5] Kim, H. S. *et al.* Nanoscale Superconducting Honeycomb Charge Order in $IrTe_2$. *Nano Lett.* **16**, 4260–4265 (2016).

[6] Ko, K.-T. *et al.* Charge-ordering cascade with spin-orbit Mott dimer states in metallic iridium ditelluride. *Nat. Commun.* **6**, 7342 (2015).

[7] Yoshida, M., Kudo, K., Nohara, M. & Iwasa, Y. Metastable Superconductivity in Two-Dimensional $IrTe_2$ Crystals. *Nano Lett.* **18**, 3113–3117 (2018).

[8] Morita, M. & Okuma, S. Magnetoresistance and vortex states below the 2D superconductor–insulator transition near $T = 0$. *Physica C: Superconductivity* **392**, 406–409 (2003).

[9] Sharma, C. H., Surendran, A. P., Varma, S. S. & Thalakulam, M. 2D superconductivity and vortex dynamics in $1T$-MoS_2. *Communications Physics* **1**, 1–8 (2018).

[10] Paradiso, N., Nguyen, A.-T., Kloss, K. E. & Strunk, C. Phase slip lines in superconducting few-layer $NbSe_2$ crystals. *2D Materials* **6**, 025039 (2019).

[11] You, J. S. *et al.* Thermoelectric properties of the stripe-charge ordering phases in $IrTe_2$. *Physical Review B* **103**, 045102 (2021).

[12] Tinkham, M. *Introduction to Superconductivity* (Dover Publications, Inc., New York, 2004).

[13] Kim, M., Kozuka, Y., Bell, C., Hikita, Y. & Hwang, H. Y. Intrinsic spin-orbit coupling in superconducting δ-doped $SrTiO_3$ heterostructures. *Phys. Rev. B* **86**, 85121 (2012).

[14] Tinkham, M. Effect of Fluxoid Quantization on Transitions of Superconducting Films. *Phys. Rev.* **129**, 2413–2422 (1963).

[15] Talantsev, E. F. & Tallon, J. L. Universal self-field critical current for thin-film superconductors. *Nat. Commun.* **6**, 7820 (2015).

[16] Talantsev, E. F. *et al.* On the origin of critical temperature enhancement in atomically thin superconductors. *2D Mater.* **4**, 25072 (2017).

[17] Talantsev, E., Crump, W. P. & Tallon, J. L. Thermodynamic parameters of single- or multi-band superconductors derived from self-field critical currents. *Ann. Phys.* **529**, 1700197 (2017).

[18] Liu, C. *et al.* Two-dimensional superconductivity and topological states in $PdTe_2$ thin films. *Phys. Rev. Mater.* **2**, 094001 (2018).

[19] Zheliuk, O. *et al.* Josephson coupled Ising pairing induced in suspended MoS_2 bilayers by double-side ionic gating. *Nat. Nanotechnol.* **14**, 1123–1128 (2019).

[20] Wilson, M. N. *et al.* μSR and magnetometry study of superconducting 5% Pt-doped $IrTe_2$. *Phys. Rev. B* **94**, 184504 (2016).

[21] Crump, W. P. Available at `https://github.com/WayneCrump/BCS-theory-critical-current-fit` (2017).

[22] Toriyama, T. *et al.* Switching of conducting planes by partial dimer formation in $IrTe_2$. *J. Phys. Soc. Jpn.* **83**, 033701 (2014).

[23] Becker, P., Scyfried, P. & Siegert, H. The lattice parameter of highly pure silicon single crystals. *Z. Phys. B* **48**, 17–21 (1982).

[24] Ding, Y. & Xiao, B. Thermal expansion tensors, Grüneisen parameters and phonon velocities of bulk MT_2 (M= W and Mo; T= S and Se) from first principles calculations. *Rsc Advances* **5**, 18391–18400 (2015).

[25] Plechinger, G. *et al.* Control of biaxial strain in single-layer molybdenite using local thermal expansion of the substrate. *2D Materials* **2**, 015006 (2015).

[26] Nicholson, C. W. *et al.* Uniaxial strain-induced phase transition in the 2D topological semimetal $IrTe_2$. *Communications Materials* **2**, 1–8 (2021).

[27] Eliashberg, G. Interactions between electrons and lattice vibrations in a superconductor. *Sov. Phys. JETP* **11**, 696–702 (1960).

[28] Allen, P. B. & Dynes, R. Transition temperature of strong-coupled superconductors reanalyzed. *Phys. Rev. B* **12**, 905 (1975).

[29] McMillan, W. Transition temperature of strong-coupled superconductors. *Phys. Rev.* **167**, 331 (1968).

[30] Yoshida, M. *et al.* Controlling charge-density-wave states in nano-thick crystals of $1T$-TaS_2. *Sci. Rep.* **4**, 7302 (2014).

[31] Yoshida, M. *et al.* Extended Polymorphism of Two-Dimensional Material. *Nano Lett.* **17**, 5567–5571 (2017).
